# Weighted gene co-expression network indicates that the *DYNLL2* is an important regulator of chicken breast muscle development and is regulated by miR-148a-3p

**DOI:** 10.1186/s12864-022-08522-8

**Published:** 2022-04-04

**Authors:** Yuanfang Li, Pengtao Yuan, Shengxin Fan, Bin Zhai, Wenjiao Jin, Donghua Li, Hong Li, Guirong Sun, Ruili Han, Xiaojun Liu, Yadong Tian, Guoxi Li, Xiangtao Kang

**Affiliations:** 1 College of Animal Science and Technology, Henan Agricultural University, Zhengzhou, 450046 China; 2 Henan Key Laboratory for Innovation and Utilization of Chicken Germplasm Resources, Zhengzhou, 450046 China

**Keywords:** Muscle fibers, WGCNA, Chicken primary myoblasts, *DYNLL2*, miR-148a-3p

## Abstract

**Background:**

The characteristics of muscle fibers determine the growth and meat quality of poultry. In this study, we performed a weighted gene co-expression network analysis (WGCNA) on the muscle fiber characteristics and transcriptome profile of the breast muscle tissue of Gushi chicken at 6, 14, 22, and 30 weeks.

**Results:**

A total of 27 coexpressed biological functional modules were identified, of which the midnight blue module had the strongest correlation with muscle fiber and diameter. In addition, 7 hub genes were found from the midnight blue module, including LC8 dynein light chain 2 (*DYNLL2*). Combined with miRNA transcriptome data, miR-148a-3p was found to be a potential target miRNA of *DYNLL2*. Experiments on chicken primary myoblasts (CPMs) demonstrated that miR-148a-3p promotes the expression of myosin heavy chain (MYHC) protein by targeting *DYNLL2*, proving that it can promote differentiation of myoblasts.

**Conclusions:**

This study proved that the hub gene *DYNLL2* and its target miR-148-3p are important regulators in chicken myogenesis. These results provide novel insights for understanding the molecular regulation mechanisms related to the development of chicken breast muscle.

**Supplementary Information:**

The online version contains supplementary material available at 10.1186/s12864-022-08522-8.

## Background

Skeletal muscle is an important economic trait for animals. Chicken skeletal muscle is one of the most important sources of meat for humans. Multinucleated myofibers are the functional units of skeletal muscle, and the characteristics of myofibers determine the growth and development of poultry and meat quality [1]. The characteristics of muscle fibers include muscle fiber diameter and muscle fiber density. Generally, muscle fiber diameter and muscle fiber density are negatively correlated [2, 3]. The more muscle fibers per unit area, the greater the density of muscle fibers and the more tender the meat [4]. The number of muscle fibers is determined before birth. The increase in muscle after birth mainly depends on the increase in the volume of original muscle fibers [5]. The formation of muscle fibers is regulated by myoblasts. During the embryonic period, myoblasts begin to align with each other to form multinucleated myotubes, and myoblasts exit the cell cycle after fusion [1]. After incubation, the muscle-dependent stem cells (called satellite cells) of adult skeletal muscle are activated, which results in a large amount of proliferation, differentiation, and fusion to promote muscle fiber hypertrophy [6]. Muscle fiber formation is a complex trait; it is regulated by a complex network [7, 8]. Therefore, network analysis has now been proposed as a solution for systems biology research, especially research involving transcriptome data [9]. WGCNA is a new network modeling method, which was developed to analyze transcriptome profiles combined with phenotypic data and used to mine key modules and hub gene networks [10].

The formation of muscle fiber is controlled by many myogenic regulators [11, 12], involving many signaling factors of early muscle fiber in poultry and the molecular mechanism of muscle fiber formation [13, 14]. In our previous research, we found that *DYNLL2*, as a differentially expressed gene during muscle development, is highly expressed in chicken breast muscle tissue [15]. Furthermore, *DYNLL2* mainly forms a complex with myosin V motor, myosin V had an important role in regulating growth and development in Eriocheir Sinensis [16]. Moreover, it has been proposed that *DYNLL*2 negatively regulates the BH3-only class of *Bcl-2* family proteins, thereby inhibiting the pro-apoptotic activity of these proteins [17]. *DYNLL2* is involved in many cellular processes [18], but so far, there is no evidence for its role in chicken or muscle cells.

It is well known that genes are subject to extensive post-transcriptional regulation [19, 20]. MicroRNAs (miRNAs) are small non-coding RNAs that bind to the 3'-UTR of target mRNAs to inhibit their expression after gene transcription [21]. Numerous studies have proved that miRNA is an important regulator of skeletal muscle satellite cells (SMSC) [22, 23]. It shows that miR-148a is one of the most abundant miRNAs in the skeletal muscles of pigs and chickens [24, 25]. In our previous data, we also found that miR-148a-3p is highly expressed in chicken breast muscle tissue and predicted that it may target *DYNLL2* [15]. The identification of new miRNA-mRNA networks provides insight and helps to reveal the complex regulatory mechanisms of muscle growth and development. In this study, we further determined the target relationship between *DYNLL2* and miR-148a-3p. It showed that elevated expression of miR-148a-3p significantly promoted the differentiation of SMSCs in chicken, but did not affect proliferation [25]. In addition, it identifies miR-148a as a novel myogenic microRNA that mediates myogenic differentiation [26]. Therefore, we were interested to know whether the effect of miR-148a-3p on muscle cells was caused by targeting *DYNLL2*.

The current research on the regulatory network of chicken muscle development is not comprehensive. Gushi chickens are a well-known excellent breed in China [27]. This study used Gushi chickens as a model to study and improve the understanding of the muscle development regulation network. We performed WGCNA based on the transcriptome profile data of the breast muscle of Gushi chickens aged 6, 14, 22, and 30 weeks and on the phenotypic data of muscle fiber density and diameter. We wanted to find the hub genes that affect muscle fibers based on WGCNA and determine the important regulatory miRNAs of the hub gene through miRNA-mRNA association analysis. In addition, the key regulatory network between the hub gene *DYNLL2* and miR-148a-3p was also thoroughly verified at the cellular level to examine its effects on the differentiation of myoblasts. This research has further strengthened and provided new insights on the network mechanisms that regulate chicken myogenesis.

## Results

### Specific modules related to breast muscle fiber development

**Fig. 1:**
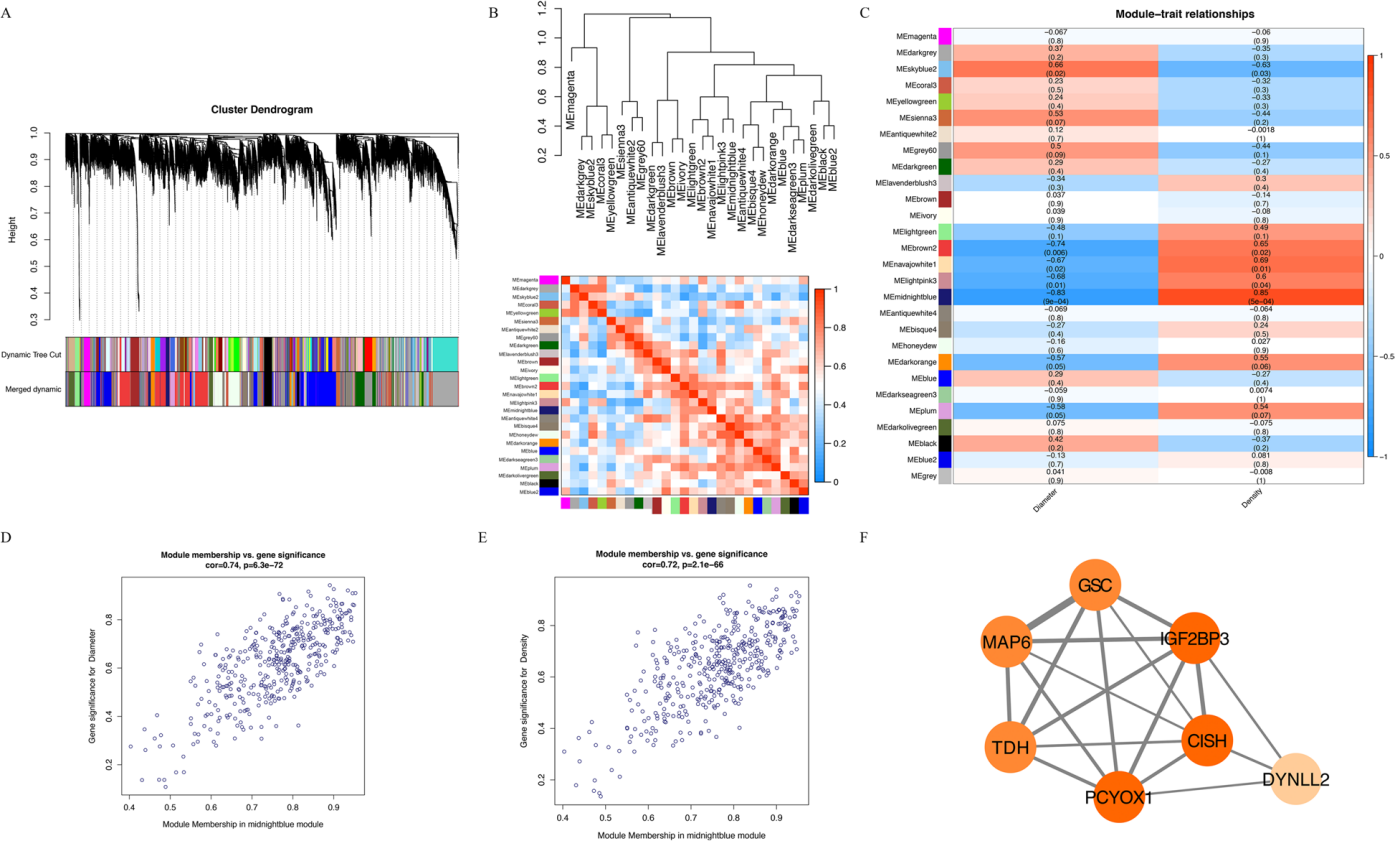
WGCNA analysis of gene expression profile with muscle fiber and diameter in Gushi chicken breast muscle tissue at 6, 14, 22, and 30 weeks. **A** The cluster dendrogram constructs the gene modules and module merging. **B** Correlation analysis between the modules. **C** Analysis of the correlation between different module genes and phenotypes. **D** GS and MM analysis for diameter. **E** GS and MM analysis for density. **F** The connection network of the 7 hub genes in the midnight blue modules (the thickness of the line between them represents the weight value between them, and the depth of the color of the node represents the number of connected nodes)

Based on the transcriptome data of the Gushi chicken breast muscle tissue at 6, 14, 22, and 30 weeks and the removal of genes with an FPKM less than 0.5, a total of 18,162 genes were obtained for WGCNA (Table S1). We performed a gene cluster tree analysis on the gene expression profiles of breast muscle tissue and identified 28 modules (the grey module will be removed in the subsequent analysis, where grey indicates that it has not been divided into any module) (Fig. 1A). Next, we conducted a correlation analysis of these 27 modules. The dendrogram combines the positively related gene modules together, and the heat map cluster analysis reflects the same results (Fig. 1B).

Among the 27 modules, we found that 5 modules (midnight blue, skyblue2, brown2, navajowhite1, and lightpink3) were significantly related to muscle fiber diameter and density (*p* < 0.05) (Table S2), of which the midnight blue module had the strongest correlation with muscle fiber and diameter (Fig. 1C). There was a significant positive correlation with muscle fiber density, with a correlation coefficient of 0.85, and a significant negative correlation with muscle fiber diameter, with a correlation coefficient of -0.83. In addition, we found that the module skyblue2, which was significantly positively correlated with muscle fiber diameter (correlation coefficient > 0.5, *p* < 0.05), was significantly negatively correlated with muscle fiber density (correlation coefficient < -0.5, *p* < 0.05). The modules with a significant positive correlation in muscle fiber density (brown2, navajowhite1, and lightpink3) had a significant negative correlation in muscle fiber diameter, which was also observed with the regular pattern of muscle development. Therefore, it can be seen from this result that these 5 modules are related to muscle development and that the midnight blue module is the most significant.

### Hub genes related to breast muscle fiber development

Through the above identification of the important modules, we found that midnight blue was the most important module. There are 261 known genes and 147 new genes in the midnight blue module (Table S2). We also performed gene significance (GS) and module membership (MM) analysis on the module to explore hub genes (Fig. 1D-E). Then, we screened the hub gene set according to |GS|>0.2 and |MM|>0.8 and obtained a total of 139 genes (*P*<0.05), of which 103 genes were known genes and 36 genes were novel genes (Table S3). In addition, we performed Kyoto Encyclopedia of Genes and Genomes (KEGG) analysis on the midnight blue module and found that only one KEGG pathway was significantly enriched (*q-*value < 0.05) (Figure S1A), namely, glycosaminoglycan degradation, and that a total of 4 genes were enriched, namely, N-sulfoglycosamine sulfohydrolase (*SGSH*), *GNS*, *β*-glucuronidase (*GUSB*) and hyaluronoglucosaminidase 1 (*HYAL1*). We also performed Gene Ontology (GO) analysis on the midnight blue module and found that the genes in that module were significantly enriched in 5 GO terms (*q-*value < 0.05) (Figure S1B), namely, intracellular, organelle, membrane-bounded organelle, intracellular organelle, and intracellular membrane-bounded organelle. We found that a total of 67 genes were enriched in these GO terms. We correlated the above 71 genes enriched in KEGG and GO terms with the differentially expressed genes between 6 weeks, 14 weeks, 22 weeks, and 30 weeks in Gushi chicken breast muscle tissue identified in our previous study (Table S4) and found that there were 10 genes in common, namely, *DYNLL2*, cytokine-inducible SH2-containing protein (*CISH*), *MAP 6*, insulin-like growth factor 2 mRNA-binding protein 3 (*IGF2BP3*), goosecoid (*GSC*), heat shock protein 70 (*hsp70*), solute carrier family 25 member 17 (*SLC25A17*), prenylcysteine oxidase 1 (*PCYOX1*), *FSD1L*, and thermostable direct hemolysin (*TDH*). Among these, we found that *DYNLL2*, *CISH*, *MAP 6*, *IGF2BP3*, *GSC*, *PCYOX1*, and *TDH* also conformed to our hub gene screening, and therefore they could be used as important hub genes for further research; their connection diagrams are shown in Fig. 1F. In addition, we found that *DYNLL2* had the highest expression in breast muscle at the different stages (Figure S1C). We speculate that it plays an important role in different stages of muscle development, so we further studied the function of *DYNLL2*.

### The *DYNLL2* was targeted by miR-148a-3p

**Fig. 2:**
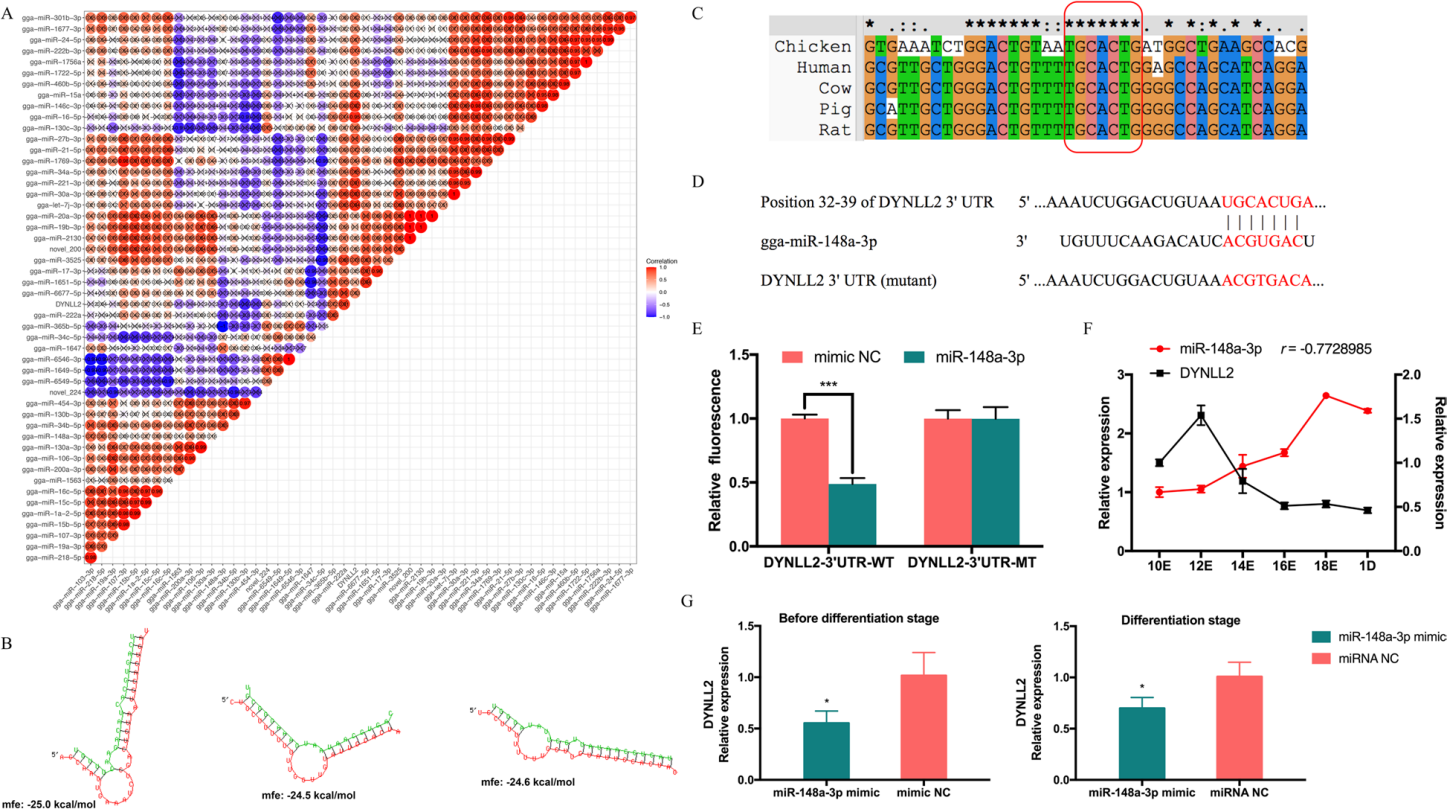
Verification of the target relationship between miR-148a-3p and *DYNLL2*. **A** Analysis of the correlation coefficient between *DYNLL2* and target miRNA expression. **B** Binding free energy analysis of *DYNLL2* and target miRNAs. **C** Conservation analysis of miR-148a-3p and *DYNLL2*-3’UTR binding sites in different species (chicken, human, cow, pig, and rat). **D** Site sequences of the wild-type vector for *DYNLL2*-3’UTR (*DYNLL2*-3’UTR-WT) and the mutant-type vector for *DYNLL2*-3’UTR (*DYNLL2*-3’UTR-MT) of the miR-148a-3p binding site. **E** The dual-luciferase reporter system assessed the binding of *DYNLL2* and miR-148a-3p in DF-1 cells. **F** Expression of *DYNLL2* and miR-148a-3p in chicken embryos from embryonic day 10 to 1 day old. r is the correlation coefficient between the expression of *DYNLL2* and miR-148a-3p. **G** Changes in *DYNLL2* after transfection with miR-148a-3p mimics in the pre-differentiation and differentiation period. All experiments were performed in triplicate, and the data are expressed as the mean ± S.E.M. (* *p* <0.05; ** *p* <0.01, *** *p* <0.001).

Our previous research showed that *DYNLL2* has abundant target miRNAs, proving that it is subject to extensive posttranscriptional regulation [15]. Therefore, we used the association analysis of mRNA and miRNA to find all the predicted target miRNAs of *DYNLL2*, and a total of 55 target miRNAs were found. Their correlation analysis with the *DYNLL2* gene is shown in Fig. 2A. The results showed that miR-103-3p, miR-19a-3p, miR-1563, miR-200a-3p, miR-106-3p, miR-130a-3p, miR-148a-3p, miR-34b-5p, miR-130b-3p, miR-454-3p, miR-6549-5p, miR-1647, and miR-34c-5p all had a negative correlation with *DYNLL2* and could be used as effective potential target miRNAs of *DYNLL2*. Among these, the negative correlation coefficient of miR-148a-3p, miR-130b-3p, miR-454-3p, and *DYNLL2* was less than -0.7; thus, the predicted target relationship between them was more reliable. After that, we used RNAhybird to predict the binding free energy of miR-148a-3p, miR-130b-3p, and miR-454-3p with *DYNLL2* (Fig. 2B). The free binding energy value of miR-148a-3p and *DYNLL2* was the lowest, which proves that the interaction between the two was stronger. Therefore, we wanted to verify the target relationship between *DYNLL2* and miR-148a-3p. We first predicted the binding sites between miR-148a-3p and *DYNLL2* on the Targetscan website, it was found that the binding sites between miR-148a-3p and *DYNLL2* 3’UTR were highly conserved among chicken, human, cow, pig, and rat (Fig. 2C). In addition, we constructed a wild-type vector of *DNYLL2*-3’UTR (*DYNLL2*-3’UTR-WT) and a mutant-type vector for *DYNLL2*-3’UTR (*DYNLL2*-3’UTR-MT) of the miR-148a-3p binding site (Fig. 2D). Recombinant psiCHECK reporter vectors (*DYNLL2*-3’UTR-WT and *DYNLL2*-3’UTR-MT)​ were cotransfected with miR-148a-3p mimics or mimics NC. We found that after transfection with miR-148a-3p mimics, compared with the control group, the fluorescence activity of *DYNLL2*-3’UTR in the wild-type group was significantly reduced, and no significant difference was observed in the *DYNLL2*-3’UTR in the mutant-type group (Fig. 2E). The dual-luciferase reporter assay showed that miR-148a-3p could directly bind to the predicted target site of *DYNLL2*-3’UTR. After that, we wanted to further explore their dynamic expression trends in CPMs and muscles. Then, we tested the expression trends of miR-148a-3p and *DYNLL2* during the embryonic period from embryonic day 10 to day 1 of CPMs. The expression trends of miR-148a-3p and *DYNLL2* were significantly negatively correlated during the embryonic stage (*r*=-0.77) (Fig. 2F). The overexpression of miR-148a-3p also inhibited the mRNA expression level of *DYNLL2* in both pre-differentiation and differentiation period (Fig. 2G). The above results show that miR-148a-3p and *DYNLL2* not only had target sites but also showed opposite expression trends during the embryonic stage of chickens.

### miR-148a-3p promotes myoblast differentiation by targeting *DYNLL2*

**Fig. 3:**
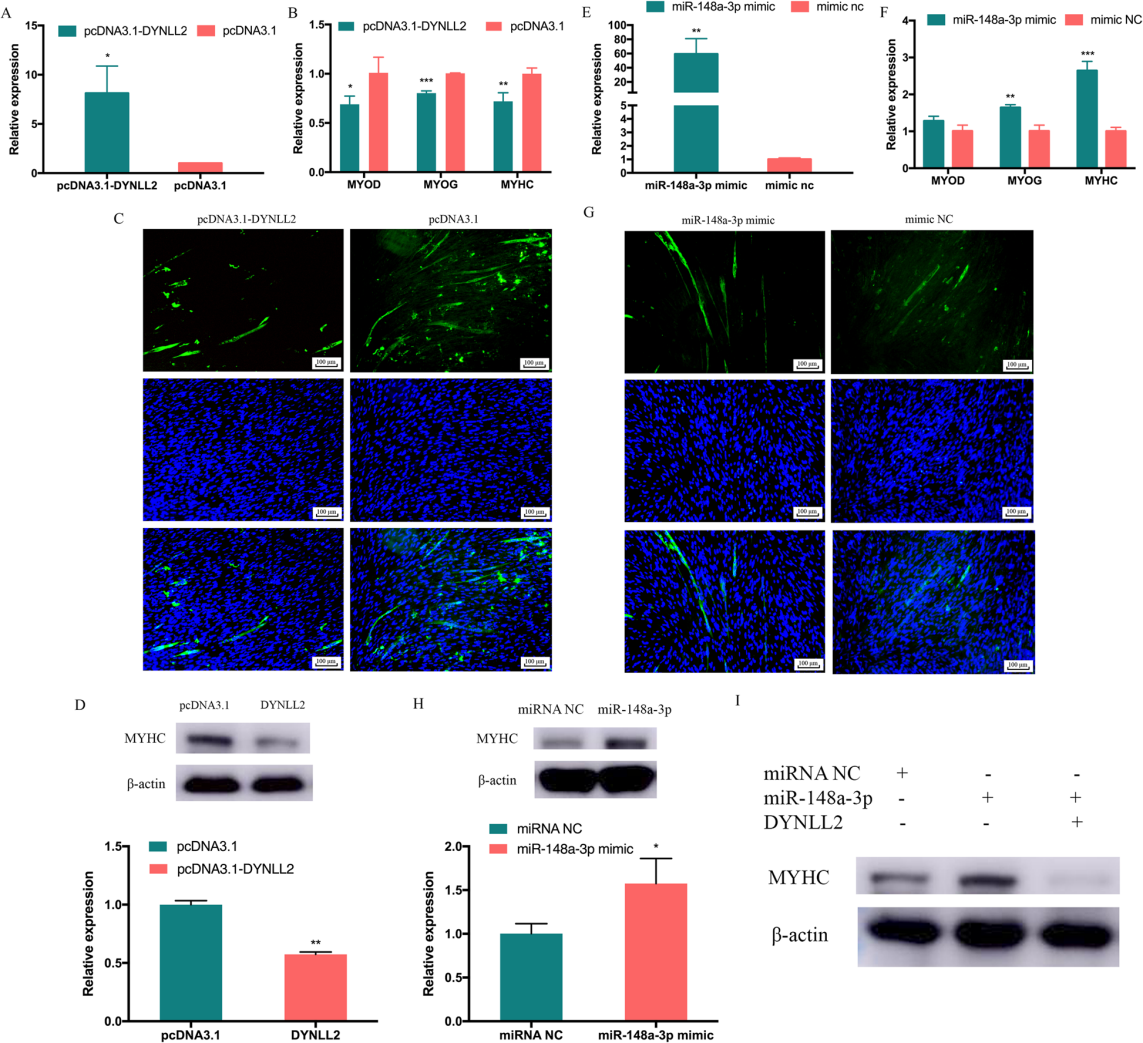
The effect of *DYNLL2* and miR-148a-3p on CPMs differentiation. **A** Relative expression of *DYNLL2* after overexpression in CPMs. **B** The changes of differentiation marker genes *MYOD*, *MYOG*, and *MYHC* after *DYNLL2* transfection in CPMs. **C** MYHC staining after transfection of *DYNLL2*. **D** Changes in the differentiation marker MYHC protein after *DYNLL2* transfection of CPMs (The order of the samples, lanes 1-2: pcDNA3.1, *DYNLL2*). **E** The relative expression of miR-148a-3p after transfection with miR-148a-3p mimics. **F** The changes of differentiation marker genes *MYOD*, *MYOG*, and *MYHC* after miR-148a-3p transfection in CPMs. **G** MYHC staining after transfection with the miR-148a-3p mimic in CPMs. **H** Changes in the differentiation marker MYHC protein after transfection with the miR-148a-3p mimics (The order of the samples, lanes 1-2: mimic NC, miR-148a-3p mimic). **I** Changes in the differentiation marker MYHC protein after co-transfection of miR-148a-3p mimics and *DYNLL2*. (The order of the samples, lanes 1-3: mimic NC, miR-148a-3p mimic, miR-148a-3p mimic+*DYNLL2*). The data are expressed as the mean ± S.E.M. (* *p* <0.05; ** *p* <0.01, *** *p* <0.001)

After proving the target relationship between *DYNLL2* and miR-148a-3p, we also wanted to reveal their role of them in cell differentiation. To study the role of *DYNLL2* in CPMs differentiation, the overexpression vectors pcDNA3.1-*DYNLL2* and si-*DYNLL2* were transfected into CPMs. Compared with the control group transfected with pcDNA3.1, *DYNLL2* in the overexpression group increased significantly (Fig. 3A). In contrast, the si-*DYNLL2* transfection group significantly reduced the expression of *DYNLL2* (Figure S2A). After overexpression of *DYNLL2*, we found that the expression levels of *MYOD*, myogenin (*MYOG*), and *MYHC*, which are differentiation marker genes, were significantly decreased (Fig. 3B). After transfection of si-*DYNLL2*, the expression of *MYOG* increased, but the expression of *MYOD* and *MYHC* did not change significantly (Figure S2B). Furthermore, immunofluorescence staining showed that after *DYNLL2* overexpression, the area of MYHC immunofluorescence decreased (Fig. 3C). Overexpression of *DYNLL2* significantly reduced the protein expression of myoblast differentiation marker genes, MYHC (Fig. 3D, Figure S3A). Overall, the above results demonstrate that *DYNLL2* can inhibit the differentiation of CPMs. Moreover, we also investigated the potential role of miR-148a-3p in CPMs differentiation. We performed miR-148a-3p overexpression and inhibition experiments in CPMs. After 48 hours of transfection with miR-148a-3p mimics, the relative expression of miR-148a-3p was assessed (Fig. 3E), and the results showed that miR-148a-3p overexpression was successful. After overexpression of miR-148a-3p, we found that *MYOG* and *MYHC* were significantly increased, and changes in *MYOD* were not significant (Fig. 3F). After successful inhibition of miR-148a-3p (Figure S2C), it was found that *MYOD* and *MYHC* were significantly decreased, and *MYOG* was not significantly changed (Figure S2D). MYHC immunofluorescence staining of CPMs showed that the MYHC fluorescence area of the miR-148a-3p mimics transfection group was more than the control group (Fig. 3G). However, compared with the control group, the expression of MYHC protein increased significantly after miR-148a-3p overexpression (Fig. 3H, Figure S3B). From the above results, it seems that *DYNLL2* and miR-148a-3p have opposite trends in myoblast differentiation, so we would like to further understand whether miR-148a-3p promotes myoblast differentiation due to targeting and reducing *DYNLL2*. Therefore, we designed a recovery experiment, we found an increase in MYHC after overexpression of miR-148a-3p, and then we found a downward trend in MYHC after co-transfection of miR-148a-3p and *DYNLL2* (Fig. 3I, Figure S3C). We also saw in the previous results that miR-148a-3p could reduce the expression of *DYNLL2* during cell differentiation (Fig. 2E). Therefore, the above results all indicate that miR-148a-3p promotes the differentiation of myoblasts by targeting *DYNLL2*.

## Discussion

Skeletal muscle formation is a complex multistage developmental process [28]. Although many genes are related to muscle development, the gene network related to muscle development has not been clearly defined. WGCNA is an excellent bioinformatics tool that can deeply assess the changes in gene expression during muscle development from the phenotypic level [29]. In this study, the previously obtained Gushi chicken breast muscle transcriptome data [15, 30] and muscle fiber phenotype data were used for WGCNA to identify the key coexpression biological functional modules and key genes, which are likely to play a key role in muscle growth and development. In our study, through gene cluster tree analysis, we classified all co-expressed genes into 27 coexpression biological functional modules, of which the midnight blue module was most significantly related to muscle fiber density. A total of 261 known genes and 147 novel genes were identified. To further understand the importance of functional modules in muscle development, KEGG and GO functional enrichment analyses were subsequently conducted. Among the 27 main coexpression biological functional modules, the midnight blue module was the most important functional module, which mainly enriched some biological processes such as intracellular, organelle, membrane-bounded organelle, intracellular organelle, and intracellular membrane-bounded organelle. Furthermore, it is particularly critical to find the hub gene among the many genes in the most important module.

After we analyzed hub genes, we found that among the most important functional modules (midnight blue module), *DYNLL2*, *CISH*, *MAP 6*, *IGF2BP3*, *GSC*, *PCYOX1*, and *TDH* were the most important genes. The interaction between these genes must have an important impact on muscle development. The *CISH* gene, a well-established *GH* target gene, is increased by *GH* in bovine myoblasts. *GH* stimulates skeletal muscle growth in cattle in part through stimulation of protein synthesis in the muscle [31]. This demonstrates the presence of *MAP 6* transcripts and proteins in skeletal muscle. Deletion of *MAP 6* results in a large number of muscle modifications [32]. *IGF2BP3*, which is a translational activator of *IGF2*, is involved in the growth of skeletal muscle [33]. *GSC* is the earliest dorsal mesoderm gene, which is strongly induced by Eomes in the abdominal mesoderm and changes the cell fate to muscle and notochord [34]. There is a direct linear relationship between myosin ATPase activated by actin in vitro and *TDH* in vivo [35]. The functions of the newly identified gene *PCYOX1* in muscle development have not yet been studied. Another gene, *DYNLL2*, is a differential gene that we previously identified in different developmental stages of breast muscle tissue in Gushi chicken [15], which is highly expressed in muscle tissue and has attracted our attention.

*DYNLL*, as a conserved homodimeric eukaryotic hub protein, has been identified as having dozens of binding partners and interacts with binding partners to participate in a wide range of cellular functions [36]. Our previous studies have shown that *DYNLL2* is enriched in GO terms such as skeleton muscle hypertrophy and myosin complex [37], but the effect of *DYNLL2* on chicken muscle development is unclear. *MYOD* and *MYOG* belong to a family of myogenic regulatory factor (MRF) that drive muscle gene expression during myogenesis [38]. During somite formation, *myf-5* is expressed first, followed by myocyte enhancer factor 2 C (*MEF2C*) and *MYOD*, then *MYOG* and myocyte enhancer factor 2A (*MEF2A*), *MYHC* is finally expressed [39]. Therefore, in this study, we selected *MYOD*, *MYOG*, and *MYHC* as monitoring changes in the level of myoblast differentiation. *MYHC* is expressed in late and terminal differentiation, serves as a differentiation marker for the development of skeletal muscle and rhabdomyosarcoma, so we also examined changes in its protein levels [40]. We found that overexpression of *DYNLL2* reduced the expression of *MYOD*, *MYOG*, and *MYHC* mRNA levels. In addition, it can reduce the expression of MYHC protein, which was also confirmed by MYHC immunofluorescence experiments. These results demonstrate that *DYNLL2* can reduce the differentiation of myoblasts. Therefore, our study demonstrates that *DYNLL2* is involved in the myogenic process of myoblast differentiation.

We know that mRNAs are involved in extensive posttranscriptional regulation. miRNAs are small non-coding RNAs of approximately 22 nucleotides and are increasingly considered to be effective posttranscriptional regulators of gene expression [41]. Therefore, studying the network between genes and miRNAs is also very important for understanding gene expression. To explore the networks of *DYNLL2*-miRNAs, according to our previous combined analysis of mRNA and miRNA of Gushi chicken breast muscle, we showed that miR-148a-3p, miR-130b-3p, and miR-454-3p had a high potential for targeting *DYNLL2*. miR-130b-3p negatively regulates the expression of *RB1CC1* and affects myogenic differentiation [42]. Many studies have shown that miR-454-3p is involved in the proliferation, invasion, and apoptosis of cancer cells [43, 44]. Notably, it has been proven that miR-148a-3p is one of the most abundant miRNAs in chicken skeletal muscle [25]. Moreover, our previous study found that the expression of miR-148a-3p and *DYNLL2* showed opposite trends in breast muscle tissues of Gushi chickens at different weeks of age [15]. The free binding energy of miR-148a-3p and *DYNLL2* was the lowest, showing the possibility of strong binding. Therefore, we verified the *DYNLL2*-miR-148a-3p network. The results of our dual-luciferase reporting experiments did indicate that they have a target relationship. The results also showed that overexpression of miR-148a-3p could significantly reduce the expression of *DYNLL2*. These results all proved that *DYNLL2* was a direct target gene of miR-148a-3p. Many studies have shown that miR-148a-3p can affect cell differentiation, it showed that miR-148a-3p can be used as a candidate miRNA that affects myogenic differentiation [30]. Therefore, we were interested in whether miR-148a-3p exerted a role in myoblasts through *DYNLL2*. We examined its effect on myoblast differentiation. In our study, we found that miR-148a-3p could promote the expression of cell differentiation-related markers *MYOG* and *MYHC* at the mRNA level, and we also found that miR-148a-3p could upregulate the expression of MYHC protein. Therefore, we determined that miR-148a-3p plays a role in the differentiation of myoblasts, thereby regulating muscle development. To explore whether the promoting effect of miR-148a-3p on the differentiation of myoblasts is caused by targeting *DYNLL2*, we performed a recovery experiment. We found that overexpression of miR-148a-3p promoted MYHC protein levels, the changes were restored after adding *DYNLL2*. In addition, we also found a negative correlation between the expression of miR-148a-3p and *DYNLL2* during the embryonic stage. Therefore, these results suggest that miR-148a-3p is involved in myoblast differentiation by targeting *DYNLL2*.

## Conclusions

**Fig. 4:**
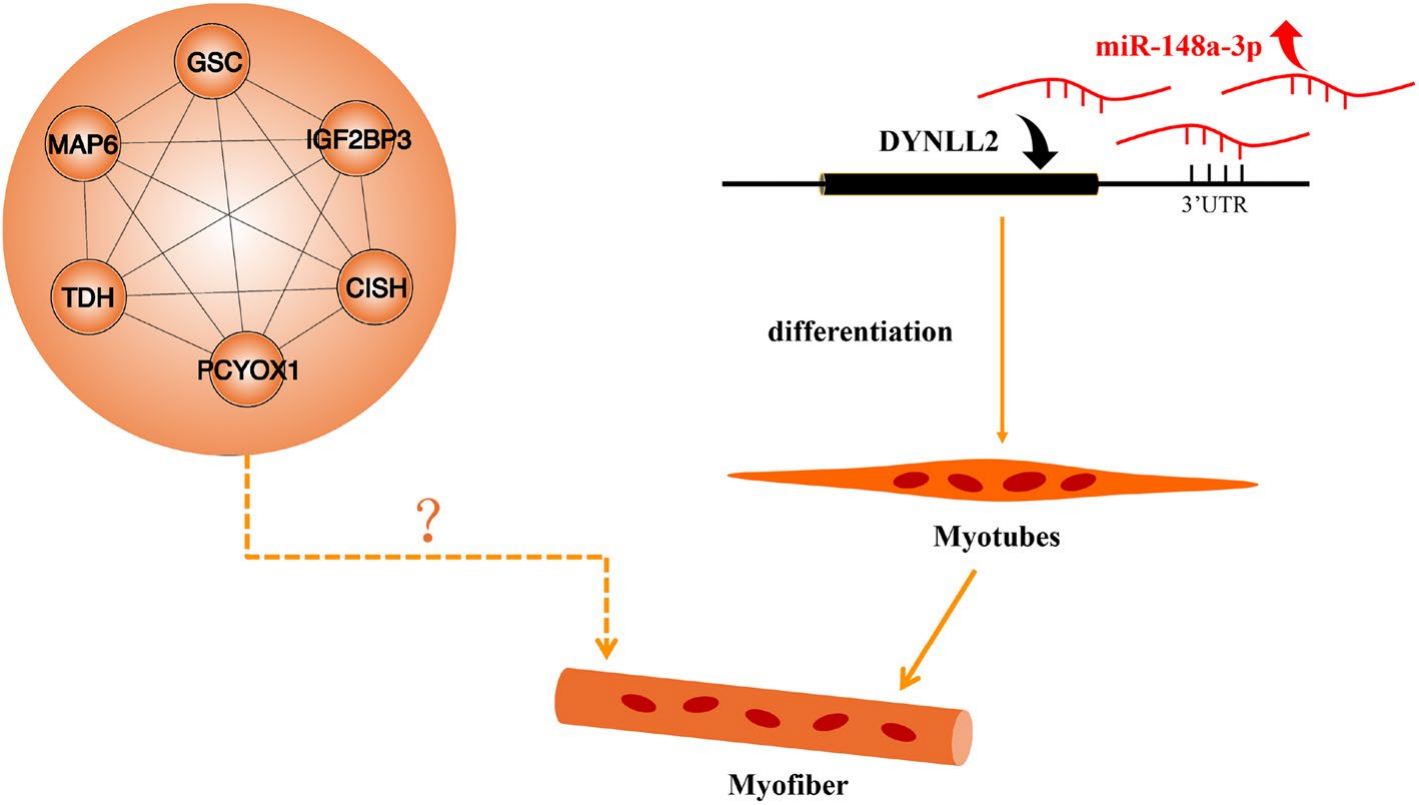
The regulatory network model involved in the effects of miR-148a-3p and *DYNLL2* and other 6 hub genes on the differentiation of CPMs. In short, miR-148a-3p inhibits the expression of *DYNLL2* mRNA by directly binding to the 3'UTR of *DYNLL2*, participates in promoting the differentiation of myoblasts. But the role of the other 6 hub genes (*CISH*, *MAP 6*, *IGF2BP3*, *GSC*, *PCYOX1*, and *TDH*) on muscle fiber formation needs to be further explored

Overall, after WGCNA analysis, we have screened genes that potentially affect the development of breast muscle fibers in chicken, namely *DYNLL2* and other six hub genes *CISH*, *MAP 6*, *IGF2BP3*, *GSC*, *PCYOX1*, and *TDH*, some of which are novel, and their role in muscle development is unclear, so further research is needed to clarify their role in muscle development (Fig. 4). In addition, we also identified that the hub gene *DYNLL2* was regulated by miR-148a-3p in chicken myoblasts to affect the differentiation of myoblasts and the process of myogenesis. This study identified many key genes and coexpression functional modules involved in muscle development, providing new and deeper insights into muscle development.

## Methods

### Data collection

We constructed 12 cDNA libraries with three biological replicates using breast muscle tissues of Gushi chicken at 6 weeks, 14 weeks, 22 weeks, and 30 weeks. The chicken breeding and selection are as described in our previous research [30]. The library was sequenced on the Illumina HiSeq 2500 platform, and a paired-end read of 150 bp was generated. Based on the length of the gene and the count of reads mapped to the gene, the fragments per kilobase per million reads (FPKM) of each gene were calculated. Then, we obtained the mRNA expression data set through the above-mentioned RNA-seq. At the same time, we also measured the muscle fiber diameter and density of the breast muscle tissues at 6 weeks, 14 weeks, 22 weeks, and 30 weeks. The average fiber diameters of the breast muscles of Gushi chickens aged 6, 14, 22, and 30 weeks were 28.46, 43.87, 49.39, and 50.85 μm, and the average density was 853.8, 350.5, 315.1, and 271.2 numbers/mm^2^, respectively. We performed the subsequent WGCNA based on RNA-seq and the muscle fiber phenotype data.

### WGCNA and module preservation

The WGCNA package in R was used to perform WGCNA [45]. After filtering the RNA-seq data to remove outliers, we deleted genes with an average FPKM value less than 0.5. We constructed a Pearson’s correlation matrix and generated a weighted adjacency matrix that emphasized strong correlations and penalizes weak correlations. Then, the appropriate β value was selected through a power calculation to generate the topological overlap matrix (TOM) [46]. The difference matrix is used to identify gene modules through the dynamic tree-cutting algorithm [47]. The branches of the tree marked with a specific color represent a module containing highly related genes. The grey part denotes background genes that do not belong to any module. We calculated the eigengene adjacency, which was based on similar coexpression in modules. The specific interaction relationship among different modules was processed through the flashClust function. A heat map was established to show the correlations of the different modules.

### Identification of key modules and hub genes

In the present study, we used two methods to identify key modules associated with the trait we were interested in. GS was calculated to quantify the relationship between genes and traits. MM was also defined to describe the correlation between ME and the gene expression profile. Moreover, module significance (MS, the average GS of all the genes in a module) was defined. Eventually, the module that was most positively correlated with the trait was regarded as the key module. Therefore, we identified the hub genes in modules through GS and MM. The gene networks were constructed by Cytoscape software (NIGMS, USA) according to the connection strength in the WGCNA module. The connection strength between each pair of nodes was calculated using the adjacency matrix aij [45].

### GO and KEGG pathway enrichment analysis

GO function and KEGG pathway analyses were performed using KOBAS 3.0 (http://kobas.cbi.pku.edu.cn/kobas3), the signaling pathway information is refered to the KEGG website (https://www.kegg.jp/kegg/pathway.html) [48]. The R language ggplot2 package was used to process the enrichment results of visualization figures. Corrected *P*-values < 0.05 were considered to be significantly enriched.

### miRNA-mRNA correlation analysis and miRNA prediction

We obtained the miRNA-mRNA interaction network based on our previous RNA-seq research on the breast muscle tissue of Gushi chicken. We use this miRNA-mRNA association analysis result as the basis for the next analysis [15]. The correlation analysis of miRNA and mRNA was analyzed by Pearson’s correlation coefficient and visualized with Hiplot (https://hiplot.com.cn/basic/cor-heatmap). The RNAhybird website (https://bibiserv.cebitec.uni-bielefeld.de/rnahybrid) was used to predict the binding free energy of miRNA and mRNA.

### Functional verification

#### Sample and cell collection

The breast muscles of 3 chicken embryos were collected at E10, E12, E14, E16, E18, and 1 day old for gene spatiotemporal expression profiling. CPMs were isolated from the leg muscle of E11 chickens. First, the muscle tissue of the leg muscles was separated from the skin and bones, placed in the culture medium, and cut with scissors. To release single cells, the muscle cell suspension was vortexed for 1 minute and then filtered through a 70 μm sieve to obtain single cells. The vortexing and filtering steps were repeated 4-6 times to obtain enough cells. Individual cells were collected by centrifugation at 500 × g. Subsequently, serial plating was performed 3 times to enrich primary myoblasts and eliminate fibroblasts. Finally, primary myoblasts were cultured in high glucose DMEM medium (HyClone, USA) with 20% FBS (Biological Industries, Israel) and 0.2% penicillin/streptomycin (Solarbio, China). All cells were cultured at 37°C in a 5% CO_2_ humidified atmosphere.

#### RNA isolation, complementary DNA (cDNA) synthesis, and quantitative real-time PCR (qRT-PCR) analysis

Total RNA was extracted from tissues or cells by TRIzol (TaKaRa, Otsu, Japan) according to the manufacturer’s instructions. The PrimeScript RT Reagent Kit with gDNA Eraser (Perfect Real Time) (TaKaRa, Japan) was used to synthesize cDNA for mRNA. The specific bulge-loop miRNA qRT-PCR primers (one RT primer and two qPCR primers for each gene) for gga-miR-148a-3p and U6 were designed by RiboBio (RiboBio, Guangzhou, China), which also supplied the mimics and inhibitor. All qRT-PCR was conducted with a LightCycler® 96 instrument qRT-PCR system (Roche, Basel, Switzerland). All reactions were run in triplicate, and data analyses were performed using the 2^-△△Ct^ method. The qRT-PCR primer sequences are listed in Table S5.

#### Plasmid construction and dual-luciferase reporter assay

Amplify the full-length CDS sequence of *DYNLL2* from chicken breast muscle cDNA, and use *Hind III* and *EcoR I* restriction enzyme sites to construct an overexpression plasmid using pcDNA3.1 vector (Promega, Madison, WI, USA). For the construction of the psiCHECK^TM^-2 dual-luciferase reporter vector (Promega, Wisconsin, USA), we first predicted the binding sites between miR-148a-3p and *DYNLL2* on the Targetscan website (https://www.targetscan.org/) [49], the wild-type sequences of the *DYNLL2* 3’UTR that contained the putative gga-miR-148a-3p binding sequence TGCACTG were amplified by PCR using chicken breast muscle cDNA, then use *Xho I* and *Not I* restriction enzyme sites to construct the wild-type sequence on the psiCHECK ^TM^-2 vector. The mutant-type sequences of the *DYNLL2* 3’UTR were generated by changing the binding site of gga-miR-148a-3p from TGCACTG to ACGTGAC. The primer sequences are listed in Table S5. To investigate the binding sites of the *DYNLL2* 3’UTR with miR-148a-3p, DF-1 cells were cotransfected with the *DYNLL2*-WT or the *DYNLL2*-MUT reporter plasmids individually, in combination with the mimics NC or miR-148a-3p mimics, respectively. At 48 h posttransfection, firefly and renilla luciferase activities were measured by a Dual-Luciferase Reporter Assay System (Promega, USA) following the manufacturer’s instructions.

#### Immunofluorescence

For immunofluorescence, cells were seeded in 24-well plates. After transfection, the cells were fixed in 4% paraformaldehyde at room temperature for 20 minutes, treated with 0.1% Triton X-100 for 20 minutes, and then blocked with goat serum for 30 minutes. The cells were then incubated with an anti-MYHC antibody (DHSB, USA; B103; 5 ug/ml) at 4°C overnight. The cells were treated with an anti-mouse IgG FITC-conjugated antibody (Bioss, Beijing, China; bs-0296G-FITC; 1:100), then incubated for 1 hour in the dark. The nuclei were stained with DAPI (Bioss, Beijing, China). After each step, the cells were washed with PBS 1-3 times, and images were acquired with a fluorescence microscope (Olympus, Japan).

#### Western blotting assay

The cells were lysed in lysis buffer (EpiZyme, Shanghai, China). Total cellular protein was separated using 12% SDS-PAGE and then transferred to PVDF membranes (Millipore, MA, USA). To save antibodies and ensure the consistency of the results, we cut a whole piece of PVDF membrane to facilitate subsequent incubation of MYHC and anti*-*β-actin antibodies separately. The membranes were blocked with 5% skim milk in tris -buffered saline plus 0.5% tween-20 and placed on a decolorizing shaker for 1 h. Then, the membranes were incubated with anti-MYHC (DHSB, USA; B103) and anti-β-actin (Proteintech, Wuhan, China) antibodies separately at 4 °C overnight and then incubated using a second antibody conjugated to HRP (Proteintech, Wuhan, China) for 1 h at room temperature. ECL solution (EpiZyme, Shanghai, China) was used to enhance the signals. Images were obtained using Odyssey FC (LI-COR, USA). The β-actin was used to normalize protein expression. Each experiment has at least two or more biological replicates. The gray value of each band is calculated by Image J software (NIH, Bethesda, MD, USA).

#### Statistical analysis

Statistical analysis was performed using GraphPad Prism 7.0 software (San Diego, CA, USA). The results are expressed as the mean ± S.E.M. The t-test was used for statistical analysis between different groups. For all analyses, one asterisk, two asterisks, and three asterisks represent *P* <0.05, *P* <0.01, and *P* <0.001, respectively.

## Supplementary Information

**Additional file 1: Figure S1.**

**Additional file 2: Figure S2.**

**Additional file 3: Figure S3.**

**Additional file 4: Table S1.** Gene list analyzed by WGCNA.

**Additional file 5: Table S2.** Gene list of the 5 modules (midnight blue, skyblue2, brown2, navajowhite1, and lightpink3).

**Additional file 6: Table S3.** List of hub genes indicated in the manuscript with midnight blue module.

**Additional file 7: Table S4.** List of differentially expressed genes in breast muscle of Gushi chickens among 6, 14, 22, and 30 weeks.

**Additional file 8: Table S5.** Primer list.

## Data Availability

The authors declare that the data supporting the findings of this study are available within the article and its supplementary information files. All the raw sequences have been deposited in the NCBI database Sequence Read Archive with the accession numbers PRJNA516810 and PRJNA516961.

## Abbreviations

WGCNA: Weighted gene correlation network analysis
DYNLL2: LC8 dynein light chain 2
CPMs: Chicken primary myoblasts
MYHC: Myosin heavy chain
miRNAs: microRNAs
SMSC: Skeletal muscle satellite cells
GS: Gene significance
MM: Module membership
KEGG: Kyoto Encyclopedia of Genes and Genomes
SGSH: N-sulfoglycosamine sulfohydrolase
GUSB: β-glucuronidase
HYAL1: Hyaluronoglucosaminidase 1
GO: Gene Ontology
CISH: Cytokine-inducible
SH2: Containing protein
IGF2BP3: Insulin-like growth factor 2 mRNA-binding protein 3
GSC: Goosecoid
hsp70: heat shock protein 70
SLC25A17: Solute carrier family 25 member 17
PCYOX1: Prenylcysteine oxidase 1
TDH: Thermostable direct hemolysin
MYOG: Myogenin
MEF2C: Myocyte enhancer factor 2 C
MEF2A: Myocyte enhancer factor 2A
