# Supplementary figures and images for: Weighted gene co-expression network indicates that the DYNLL2 is an important regulator of chicken breast muscle development and is regulated by miR-148a-3p
Source: BMC Genomics. 2022 Apr 4;23:258. doi: 10.1186/s12864-022-08522-8 (PMC8978428; doi:10.1186/s12864-022-08522-8)

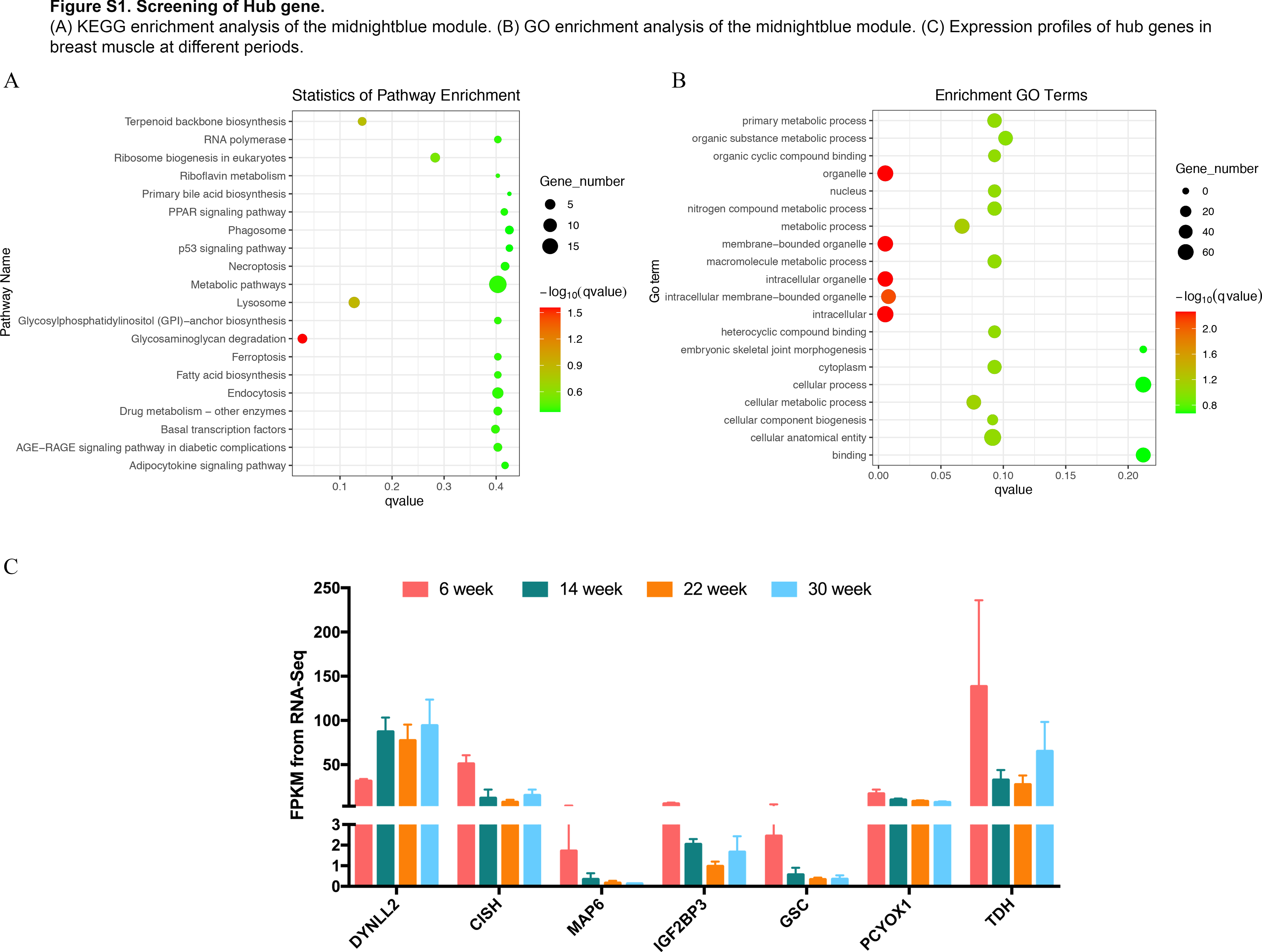

Supplement: Supplementary file 1 — Additional file 1: Figure S1. [file 12864_2022_8522_MOESM1_ESM.tif]

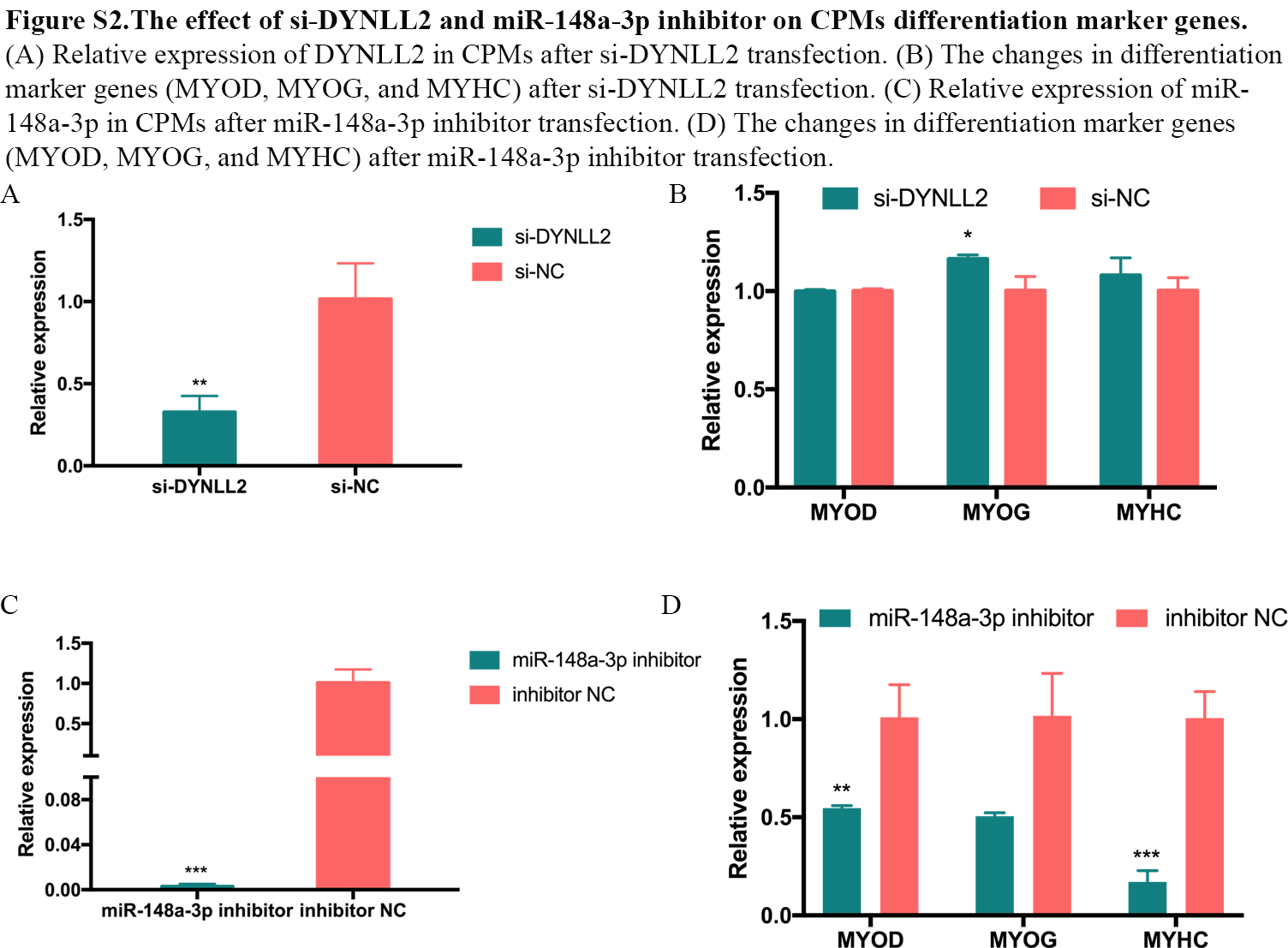

Supplement: Supplementary file 2 — Additional file 2: Figure S2. [file 12864_2022_8522_MOESM2_ESM.tif]

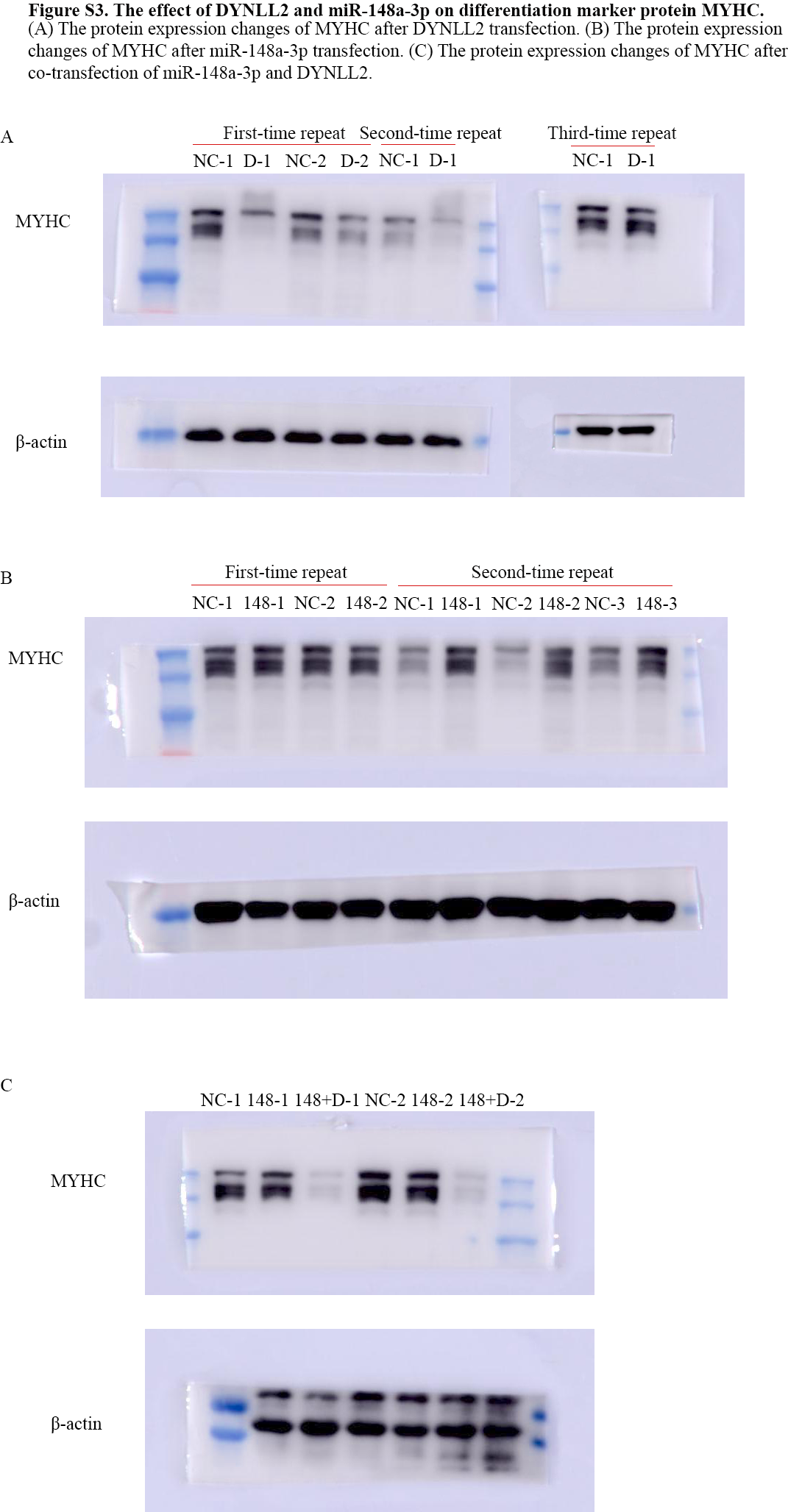

Supplement: Supplementary file 3 — Additional file 3: Figure S3. [file 12864_2022_8522_MOESM3_ESM.tif]
