# Supplementary material for: Weighted gene co-expression network indicates that the DYNLL2 is an important regulator of chicken breast muscle development and is regulated by miR-148a-3p
Source: BMC Genomics. 2022 Apr 4;23:258. doi: 10.1186/s12864-022-08522-8 (PMC8978428; doi:10.1186/s12864-022-08522-8)
Supplement: Supplementary file 8 — Additional file 8: Table S5. Primer list. [file 12864_2022_8522_MOESM8_ESM.docx]

**Table S5. Primer list.**

| **Target** | **Primer** | **Sequence（5’-3’）** |  | **AT(**°**C)** | **AE** |
| --- | --- | --- | --- | --- | --- |
| DYNLL2-CDS | F | CTCAAGCTTGCCACCATGGAGATGTCTGACAGAAAGGCTGTCATCAAGAATG |  | 56 |  |
|  | R | GGAATTCTCCAGACTTGAAGAGAAGAATTGCAACCTGACCCAAGTA |  |  |  |
| DYNLL2-WT | F | CCGCTCGAGGCAAGTGAAATCTGGACTGTAA |  | 56 |  |
|  | R | ATAAGAATGCGGCCGCTCAAAGAAGGAATTACCACTGA |  |  |  |
| DYNLL2-MT | F | CCGCTCGAGGCAAGTGAAATCTGGACTGTAAACGTGACATGGCTGAAG |  | 56 |  |
|  | R | ATAAGAATGCGGCCGCTCAAAGAAGGAATTACCACTGA |  |  |  |
| DYNLL2 (qRT-PCR) | F | TGCAACCTGACCCAAGTAAAA |  | 60 | 92.99% |
|  | R | AGGCAATGGAGAAGTACAACA |  |  |  |
| GAPDH (qRT-PCR) | F | GAACATCATCCCAGCGTCCA |  | 60 | 94.15% |
|  | R | CGGCAGGTCAGGTCAACAAC |  |  |  |

Abbreviation: AT refers to the annealing temperature; AE refers to amplification efficiency; F and R refer to the forward and reverse primers, respectively.
